# Sustainable behavioral change related to environmental sanitation in India: Issues and challenges

**DOI:** 10.4103/0019-5278.75701

**Published:** 2010

**Authors:** S. Ganesh Kumar, Sitanshu Sekhar Kar

**Affiliations:** Department of PSM, JIPMER, Pondicherry, India

Dear Sir,

Some issues related to sanitation failure in India have already been discussed.[1] There has been considerable debate and discussion as to which methodologies really work and actually achieve sustainable behavior change in developing countries like India. This is because of the problems in dealing with poor, illiterate and semi-literate rural communities in India, who continue to bear such heavy burden of disease, majority of which are preventable. It is also important at this stage because only 5 years remain to achieve the Millennium Development Goals.

Clean water, safe removal of excreta, and personal hygiene are three key elements of any strategy to improve public health. In India, majority of the population go to the open fields for defecation. It has been shown that behavior intervention related to water, sanitation and hygiene among mothers and children will result in positive increase between baseline and post intervention surveys.[2] Cost-effectiveness analysis indicates that some water supply and sanitation interventions are highly cost-effective for the control of diarrhea among under–5-year-olds, on a par with oral rehydration therapy.[3] These are relatively inexpensive interventions such as hygiene education, social marketing of good hygiene practices, regulation of drinking water, and monitoring of water quality. Recent surveys in India have indicated improved sanitation coverage in 2008 to be 31% as against 18% in 1990. Similarly, improved water supply coverage in 2008 has been reported as 88% as against 72% in1990.[4] The MDG target measures coverage but does not consider the quality of these sanitation facilities. Besides, MDG target does not measure increase in knowledge and good practice related to personal hygiene.

Motivation model of health education includes stages of interest, evaluation and decision making. The individual evinces interest in the subject and may seek more detailed information about the usefulness, limitations or applicability of new idea or practice. The subject then evaluates the social, psychological and economic aspects of the information received, and then finally decides after interpersonal communications whether to accept the new idea or proposal. Then, the acquired behavior becomes part of his/her own existing values. But the motivation model ignored the fact that it is not the individual who needs to be changed but the social environment which shapes the behavior of the individual and community. It is often found that people will not readily accept and try something new until it has been approved by the group to which they belong. Thus, behavioral change is based on precise knowledge of human ecology and understanding of the interaction between the cultural, biological, physical and social environmental factors.[5] Human behavior is affected by external factors that include skill development, accessibility of services, policy, cultural factors and internal factors like perceived social norms, perceived consequences related to their knowledge and attitude. Besides, felt needs of people are often different from the technologies that governments and NGOs supply, leading to the presence of sanitation facilities that are unused.

Breaking the feco-oral disease transmission route is the first vital step toward overcoming preventable diseases. Simple knowledge transfer on whatever methodology is employed does not automatically result in changed or improved behavior. There is growing consensus that to achieve behavior change in hygiene and sanitation practices in communities, both rural and high-density periurban, they need to be supported in ways that will stimulate social cohesion and result in group decisions being taken. Such cohesion and the building of social capital can ensure that peer pressure comes to bear, and poor hygiene practices can thus be challenged.

Some sanitation projects have been unsuccessful because they have been based on a poor understanding of what influences the behavior change that is needed to ensure good sanitation. Research suggests that the provision of subsidized latrines does not often result in improved sanitation and hygiene. On the other hand, investment in community mobilization and education has produced better results. This has encouraged people to want latrines and to improve hygiene practices. Thus, health education should be directed with direct discussion, group discussion, demonstration, use of visual aids with service facilities. This should be followed by monitoring and periodic evaluation of the public health system related to these issues so that interventions can be done appropriately.

## References

[1] Ganesh Kumar S, Jayarama S (2009). Issues related to sanitation failure in India and future perspective. Indian J Occup Environ Med.

[2] Nanan D, White F (2002). Water, sanitation, and hygiene evaluation issues. Bull World Health Organ.

[3] Varley RC, Tarvid J, Chao DN (1998). A reassessment of the cost-effectiveness of water and sanitation interventions in programmes for controlling childhood diarrhoea. Bull World Health Organ.

[4] Water supply and sanitation status in the South East Asian region.

[5] Park K, Park K (2007). Communication for health education. Park’s Text book of preventive and social medicine.

